# Overcoming Therapeutic Resistance in Triple-Negative Breast Cancer: Targeting the Undrugged Kinome

**DOI:** 10.3390/ijms27010450

**Published:** 2025-12-31

**Authors:** Chang Hoon Lee, Tuan Minh Nguyen, Yongook Lee, Seoung Gyu Choi, Phuong Ngan Nguyen, Jung Ho Park, Mi Kyung Park

**Affiliations:** 1 BK21 FOUR Team and Integrated Research Institute for Drug Development, College of Pharmacy, Dongguk University, Goyang 10326, Republic of Korea; 2 Department of Biomedical Science, Hwasung Medi-Science University, Hwaseong-si 18274, Republic of Korea

**Keywords:** triple-negative breast cancer, drug resistance, undrugged kinome, PTK7, PROTAC, antibody-drug conjugates

## Abstract

Triple-Negative Breast Cancer (TNBC) remains the most aggressive breast cancer subtype, characterized by profound heterogeneity and a lack of effective targeted therapies. Although cytotoxic chemotherapy is the standard of care, the rapid emergence of resistance driven by cancer stem cells (CSCs), metabolic plasticity, and the tumor microenvironment limits long-term survival. This review highlights the paradigm shift in TNBC treatment from 2021 to 2025, moving beyond broad cytotoxicity to precision medicine. We first examine the limitations of earlier targeted therapies, such as PI3K/AKT/mTOR inhibitors, which failed due to compensatory feedback loops and toxicity. We then discuss emerging synthetic lethality strategies targeting the G2/M checkpoint (WEE1, ATR) and mitotic kinases (PLK1, TTK) to exploit genomic instability in TP53-mutant tumors. Furthermore, we explore how novel modalities like PROTACs and Antibody–Drug Conjugates (ADCs) are unlocking the “undrugged kinome,” including targets like TNIK, PTK7, and PAK4, which were previously inaccessible. Finally, we propose that future success lies in combinatorial strategies integrating these next-generation kinase inhibitors with ADCs and immunotherapies to dismantle therapeutic resistance.

## 1. Introduction

Triple-Negative Breast Cancer (TNBC) remains the most clinically challenging subtype of breast cancer due to its profound molecular heterogeneity and the absence of standard therapeutic targets. TNBC is characterized by a high proliferation rate and a predilection for early visceral metastasis to the lungs, liver, and brain [1]. Epidemiologically, it exhibits disproportionately high incidence and mortality rates among women under the age of 40 and those of African and Hispanic ancestry [2]. The period from 2021 to 2025 has been a tumultuous era for the molecular understanding and therapeutic strategizing of TNBC. Novel risk factors, including BRCA1 germline mutations and metabolic dysregulation, have been elucidated [3], and precision medicine approaches based on the Lehmann classification have continued to evolve [4]. During this time, researchers have come to realize that improving survival rates requires moving beyond simple cytotoxicity to specifically targeting the drug-resistant cell populations that survive chemotherapy and drive recurrence.

## 2. Clinical and Biological Landscape of TNBC

### 2.1. Epidemiology and Risk Factors

TNBC, characterized by the absence of estrogen receptor, progesterone receptor, and human epidermal growth factor receptor 2 (HER2) expression, represents approximately 10–15% of all invasive breast cancers worldwide, with variations by region and population. Despite comprising a minority of cases, TNBC is disproportionately aggressive, exhibiting higher rates of recurrence, metastasis, and mortality compared to other subtypes, with 5-year survival rates often below 80% for early-stage disease and dropping to around 32% for distant metastases. According to the American Cancer Society’s Breast Cancer Facts & Figures 2024–2025 report, TNBC accounts for about 10% of breast cancers overall in the US but rises to 19% among Black women and 12–24% among Hispanic women, underscoring stark racial and ethnic disparities. These disparities are particularly pronounced in women under 40 years of age, where TNBC incidence is elevated relative to hormone receptor-positive subtypes, and in populations of African and Hispanic ancestry, reflecting a multifaceted interplay of socioeconomic stressors—such as limited access to screening and healthcare—alongside inherent biological vulnerabilities like genetic predispositions and tumor microenvironment differences [5,6]. Recent cohort studies have further linked chronic social adversity, including economic hardship and environmental exposures, to a 1.5–2-fold increased odds of TNBC diagnosis among Black women, amplifying these inequities [7,8,9].

A landmark meta-analysis by Kumar et al. (2024), synthesizing data from 33 studies involving nearly 11,000 TNBC cases, has illuminated the subtype’s distinctive risk profile, revealing strong associations with BRCA1 germline mutations (odds ratio [OR] 2.5–3.0), elevated breast density (OR 1.55), and prolonged oral contraceptive use exceeding 5 years (OR 1.32), factors that confer heightened susceptibility independent of overall breast cancer risks [3]. Notably, family history of breast cancer emerged as a significant predictor (OR 1.8), underscoring hereditary components, while protective elements like breastfeeding—reducing risk by up to 20% for hormone receptor-negative cancers—offer modifiable avenues for prevention. This profile starkly contrasts with hormone receptor-positive breast cancers, where nulliparity and early menarche predominantly drive risk; in TNBC, parity shows no protective effect, and common factors like menopausal hormone therapy or alcohol consumption do not elevate odds, highlighting the need for tailored screening and risk assessment strategies [3]. Emerging evidence also implicates obesity and metabolic syndrome as potential contributors in premenopausal women, though these require further validation in diverse cohorts to address ongoing disparities [3,6].

### 2.2. Pathophysiology and Lehmann Molecular Subtypes

The pathophysiology of TNBC differs fundamentally from that of other breast cancer subtypes. This disease is characterized by extreme genomic instability, with TP53 mutations detected in 80–90% of patients—a frequency that stands in stark contrast to the 20–30% observed in hormone receptor-positive breast cancers [10]. Although germline or somatic BRCA1/2 mutations and homologous recombination deficiency are present in only 15–20% of all TNBC cases, defects in broader DNA repair mechanisms are nearly universal. Recurrent molecular aberrations such as MYC amplification, PTEN loss, and PIK3CA mutations dramatically accelerate cell proliferation, confer immune evasion capabilities, and drive early distant metastasis, particularly to the brain and lungs [5,11].

**Figure 1:**
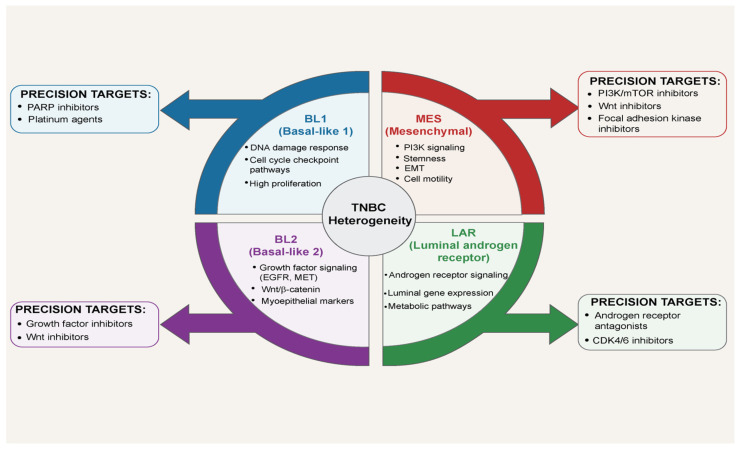
Molecular Subtypes of Triple-Negative Breast Cancer and Precision Therapeutic Targets. TNBC demonstrates molecular heterogeneity across four primary subtypes. BL1 tumors exhibit DNA damage response deficiencies and high proliferation, targetable with PARP inhibitors and platinum agents. BL2 (Basal-like 2) is characterized by growth factor signaling (EGFR, MET) and Wnt/β-catenin activation, with therapeutic options including growth factor and Wnt inhibitors. MES subtype shows PI3K signaling, stemness, and EMT features, responsive to PI3K/mTOR inhibitors, Wnt inhibitors, and FAK inhibitors. LAR demonstrates androgen receptor dependency and luminal characteristics, amenable to androgen receptor antagonists and CDK4/6 inhibitors. Arrows indicate subtype-specific precision therapeutic strategies based on molecular characteristics.

The recognition that TNBC is not a single entity but a highly heterogeneous collection of diseases was systematically established by Lehmann and colleagues in 2011. Through gene expression profiling of 587 TNBC tumors, they initially identified six molecular subtypes. Subsequent refinement by the same group in 2016 and large-scale validation in cohorts from Fudan University Shanghai Cancer Center and MD Anderson have now converged on four major tumor-intrinsic subtypes [4,11,12]. The basal-like 1 (BL1) subtype exhibits hyperactivation of cell-cycle and DNA-damage response pathways, rendering it the most sensitive to platinum agents and PARP inhibitors. The basal-like 2 (BL2) subtype is dominated by growth factor signaling and glycolysis pathways and occasionally displays squamous differentiation. The mesenchymal (MES) subtype is characterized by strong epithelial–mesenchymal transition and stem-cell features, with predominant activation of TGF-β, Wnt/β-catenin, and PI3K/AKT/mTOR signaling, leading to high chemoresistance but potential vulnerability to inhibitors of these pathways. The luminal androgen receptor (LAR) subtype, despite being estrogen receptor-negative, strongly expresses the androgen receptor and harbors PIK3CA mutations in more than half of cases, making it the only subtype that has shown clinical responses to androgen receptor antagonists and PI3K inhibitors [12,13] (Figure 1).

The previously designated immunomodulatory (IM) subtype was later found to reflect tumor-infiltrating lymphocytes and stromal components rather than intrinsic tumor cell biology and has therefore been reclassified as a microenvironmental rather than tumor-intrinsic feature [14]. More recent efforts have further subdivided the basal-like group according to immune activation status into immune-activated and immune-suppressed categories, with the immune-activated subset exhibiting high PD-L1 expression and emerging as a key population likely to benefit from immune checkpoint inhibitors [11,15].

Therefore, triple-negative breast cancer can no longer be regarded as a single disease amenable to uniform cytotoxic chemotherapy. Because each molecular subtype relies on entirely distinct survival signaling pathways, subtype-specific precision medicine strategies—DNA-damaging agents for BL1, androgen receptor blockade for LAR, PI3K/mTOR inhibitors for MES, and immune checkpoint inhibitors for immune-inflamed tumors—are now imperative. This molecular taxonomy provides the definitive scientific foundation for a paradigm shift in the treatment of TNBC [12,15] (Figure 1).

### 2.3. Current Therapeutic Standards and Limitations

Although cytotoxic chemotherapy remains the backbone of systemic therapy for TNBC, its therapeutic index is paradoxically narrow, highlighting the urgent need for more precise, less toxic, targeted approaches.

#### 2.3.1. Cytotoxic Chemotherapy: The TNBC Paradox

For early-stage, operable TNBC, the current standard is neoadjuvant chemotherapy, typically consisting of anthracycline–cyclophosphamide followed by a taxane (AC-T), often combined with platinum agents in BRCA-mutated or high-risk cases. This approach yields pathological complete response (pCR) rates of 35–50% (significantly higher than in luminal subtypes), and achievement of pCR strongly correlates with excellent long-term outcomes [16,17].

However, the seminal observation of the so-called TNBC paradox was first clearly described in a landmark retrospective analysis of 1118 patients by Liedtke and colleagues in 2008. They demonstrated that, despite the high chemosensitivity reflected by elevated pCR rates (approximately 22% with anthracycline–taxane regimens alone, rising to 30–40% with modern intensified regimens), patients who retain residual invasive disease after neoadjuvant therapy face a dramatically worse prognosis. Recurrence risk peaks sharply within the first 3 years, with 3-year distant recurrence-free survival dropping to less than 60% in non-pCR cases, compared with over 90% in those achieving pCR [18,19].

This steep prognostic divergence underscores the biological aggressiveness of chemotherapy-resistant TNBC clones. Residual tumors are frequently enriched for cancer stem cell populations, immune-evasive phenotypes, and genomic alterations that drive rapid metastatic dissemination [20,21]. Moreover, the non-selective mechanism of conventional cytotoxics inflicts substantial collateral damage, including grade 3–4 neutropenia in up to 70% of patients, febrile neutropenia, peripheral neuropathy, cardiomyopathy, and secondary leukemias, all of which severely impair quality of life and sometimes preclude completion of planned therapy [22].

Even in the metastatic setting, sequential single-agent chemotherapy (taxanes, capecitabine, eribulin, or platinum) remains the mainstay, offering objective response rates of only 10–30% and median progression-free survival of 3–6 months [23]. Acquired multidrug resistance, driven by upregulation of efflux pumps, enhanced DNA repair, and metabolic reprogramming, rapidly emerges and constitutes one of the most common mechanisms of treatment failure.

Thus, while cytotoxic chemotherapy exploits the high proliferative fraction of TNBC, its therapeutic window is extremely limited: it cures a subset through pCR but leaves the majority facing early, aggressive relapse with few effective salvage options and at the cost of considerable toxicity.

#### 2.3.2. Immunotherapy: Challenges of the Immune-Cold TME

Immune checkpoint inhibitors (ICIs) targeting the PD-1/PD-L1 axis, particularly pembrolizumab, have become a new standard of care for first-line treatment of PD-L1-positive advanced or metastatic TNBC. The pivotal phase 3 KEYNOTE-355 trial demonstrated that pembrolizumab combined with chemotherapy (nab-paclitaxel, paclitaxel, or gemcitabine/carboplatin) significantly improved both progression-free survival and overall survival exclusively in patients with a PD-L1 Combined Positive Score (CPS) ≥ 10. In the final analysis published in 2022, patients with CPS ≥ 10 achieved a median overall survival of 23.0 months with pembrolizumab plus chemotherapy versus 16.1 months with chemotherapy alone (HR 0.73; 95% CI, 0.55–0.95; *p* = 0.0093), representing the first immunotherapy regimen to demonstrate a statistically significant and clinically meaningful survival benefit in metastatic TNBC [24].

Despite this breakthrough, the benefit remains restricted to approximately 40–45% of patients who meet the CPS ≥ 10 threshold; the remaining 55–60% of patients with CPS < 10 derive no meaningful clinical benefit, with hazard ratios near 1.0 for both PFS and OS [24,25]. Response rates in PD-L1-negative disease are correspondingly low (20–30%), and virtually negligible (<10%) in “immune-cold” tumors characterized by minimal lymphocyte infiltration.

These immune-cold tumors predominantly correspond to the MES and LAR molecular subtypes, which exhibit intrinsic immune exclusion mechanisms including dense stromal barriers, low tumor mutational burden, and immunosuppressive cancer-associated fibroblasts [11,12]. Clinically, these recalcitrant, immunologically ‘cold’ phenotypes are preferentially enriched within the MES and LAR subtypes, underscoring the imperative for precision strategies designed to remodel their intrinsically immune-excluded microenvironments. In contrast, basal-like immune-activated (BLIA) tumors with high tumor-infiltrating lymphocyte (TIL) density and PD-L1 expression are far more responsive, highlighting the critical influence of the tumor microenvironment on ICI efficacy.

Thus, while pembrolizumab has transformed the treatment landscape for a biomarker-selected subset of patients, the majority of TNBC remains refractory to single-agent checkpoint blockade, underscoring the urgent need for strategies that convert immune-cold tumors into immune-inflamed ones through rational combination therapies targeting stromal, metabolic, or alternative immune checkpoints.

#### 2.3.3. Antibody–Drug Conjugates (ADCs): A New Standard and Emerging Hurdles

ADCs represent one of the most successful examples of precision oncology in solid tumors by combining the target specificity of a monoclonal antibody with the cytotoxicity of a small-molecule payload via a chemical linker [26,27]. In TNBC, where actionable driver mutations are scarce and conventional chemotherapy remains non-selective, ADCs exploit highly expressed surface antigens to achieve therapeutic indices far superior to those of naked antibodies or free cytotoxics [28,29].

Sacituzumab govitecan (SG, Trodelvy^®^) is a first-in-class TROP2-directed ADC. TROP2 (TACSTD2) is a 35 kDa transmembrane calcium signal transducer overexpressed in more than 85% of TNBCs while showing minimal expression in normal tissues, making it an ideal ADC target [30,31]. The humanized IgG1 antibody (hRS7) is conjugated via a pH-sensitive CL2A linker to govitecan (SN-38), the active metabolite of irinotecan, at a high drug-to-antibody ratio of approximately 7.6:1 [32,33]. This hydrolyzable linker permits not only intracellular payload release but also extracellular cleavage in the acidic tumor microenvironment, thereby generating a bystander killing effect that is particularly advantageous in the heterogeneous tumor landscape of TNBC [34,35].

The pivotal phase 3 ASCENT trial, which randomized 529 heavily pretreated patients with metastatic TNBC, demonstrated unprecedented survival benefit: median progression-free survival improved from 1.7 months to 5.6 months (HR 0.41) and median overall survival from 6.7 months to 12.1 months (HR 0.48), corresponding to a 52% reduction in the risk of death compared with physician’s-choice chemotherapy [36]. Confirmatory biomarker analyses revealed consistent efficacy regardless of TROP2 expression level (H-score 0–300), BRCA status, or PD-L1 status [37,38].

Despite this success, several clinical and biological challenges remain. Grade 3 or higher neutropenia occurs in 51–63% of patients and diarrhea in 10–13%, largely driven by off-target SN-38 release and UGT1A1 polymorphism-related glucuronidation defects [36,38,39]. Resistance mechanisms include downregulation or shedding of TROP2, increased ABCG2-mediated efflux of SN-38, epithelial–mesenchymal transition, and lysosomal sequestration [28,35,40,41]. These resistance mechanisms underscore a critical bottleneck in current ADC therapeutics. Notably, antigen remodeling (e.g., TROP2 downregulation) and the acquisition of payload tolerance act as primary drivers of disease progression following an initial response, highlighting the imperative to engineer next-generation linkers and explore combinatorial strategies.

Next-generation ADCs are rapidly advancing in TNBC. Datopotamab deruxtecan (Dato-DXd), a TROP2-directed topoisomerase I inhibitor with a cleavable tetrapeptide linker, has shown promising activity [42]. Trastuzumab deruxtecan (T-DXd) has demonstrated remarkable efficacy even in HER2-low (IHC 1+ or 2+/ISH–) TNBC through its potent bystander effect across HER2-heterogeneous tumors [43,44]. Additional candidates such as patritumab deruxtecan (HER3-DXd) and ADCs targeting LIV-1, folate receptor-α, and B7-H4 are under active investigation [45,46].

## 3. The Roots of Recurrence: Mechanisms of Drug Resistance

### 3.1. Cancer Stem Cells (CSCs) and Dormancy

**Figure 2:**
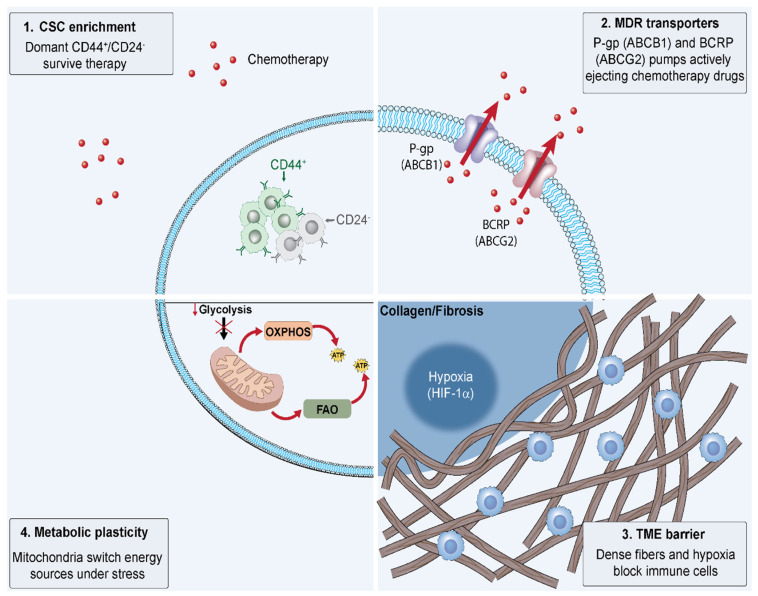
Mechanisms of Chemotherapy Resistance in Triple-Negative Breast Cancer. This schematic illustrates four distinct biological barriers that limit the efficacy of chemotherapy and immunotherapy. 1, Enrichment of Cancer Stem Cells (CSCs). While conventional chemotherapy targets rapidly dividing bulk tumor cells, quiescent CSC populations (characterized by CD44+/CD24− phenotypes) evade toxicity and survive, serving as a reservoir for tumor recurrence. 2, Efflux by MDR transporters. The upregulation of ATP-binding cassette (ABC) transporters, specifically P-glycoprotein (P-gp/ABCB1) and Breast Cancer Resistance Protein (BCRP/ABCG2), actively pumps chemotherapeutic agents out of the cytoplasm, preventing lethal intracellular drug accumulation. 3, Physical and physiological barriers in the Tumor Microenvironment (TME). Dense extracellular matrix deposition (collagen/fibrosis) physically hinders drug diffusion. Concurrently, hypoxic conditions driven by HIF-1α create an immunosuppressive niche that restricts immune cell infiltration. 4, Metabolic plasticity. Under therapeutic stress or nutrient deprivation, cancer cells demonstrate metabolic flexibility, shifting their reliance from glycolysis to mitochondrial oxidative phosphorylation (OXPHOS) and fatty acid oxidation (FAO) to sustain ATP production and survival.

The persistent challenge of therapeutic failure in TNBC stems from sophisticated drug resistance mechanisms that TNBC cells acquire through multifaceted survival adaptations, transcending mere genetic alterations and encompassing dynamic interactions at cellular, metabolic, and environmental levels. These mechanisms not only enable initial evasion of cytotoxic therapies but also foster tumor evolution toward a more aggressive, recurrent state, with recurrence rates exceeding 30% within five years post-treatment in high-risk cases [4,47]. Central to this resilience is the enrichment of CSCs, a subpopulation exhibiting enhanced self-renewal, quiescence, and therapy tolerance. In TNBC, particularly the MES subtype, CSCs are predominantly identified by the CD44+/CD24−/low phenotype, coupled with high aldehyde dehydrogenase 1 (ALDH1) activity, which confers detoxification capabilities and resistance to oxidative stress [20,48]. Conventional chemotherapeutics like taxanes and anthracyclines preferentially target proliferating bulk tumor cells, sparing these relatively dormant CSCs that rely on robust DNA repair pathways, such as enhanced homologous recombination and base excision repair, to mitigate genotoxic damage [49]. As a result, post-treatment residual tumors display a disproportionate CSC enrichment—up to 20-fold increase in CSC frequency—driving relapse with heightened metastatic potential and stemness-associated gene signatures, including SOX2 and NANOG upregulation, as evidenced in patient-derived xenografts (PDXs) from neoadjuvant chemotherapy failures [21,50]. In addition to their role in therapeutic resistance, CSCs actively orchestrate immune evasion. Emerging evidence highlights the CD24–Siglec-10 axis as a critical innate immune checkpoint; the engagement of Siglec-10 on macrophages by CD24 transduces a potent anti-phagocytic signal, thereby shielding tumor cells from clearance, particularly in PD-L1-negative TNBC phenotypes. This CSC-mediated recurrence is further exacerbated in hypoxic niches, where low oxygen levels stabilize hypoxia-inducible factor 1α (HIF-1α), promoting CSC maintenance and epithelial-mesenchymal transition (EMT) via Twist and Snail transcription factors [51] (Figure 2).

### 3.2. Multidrug Resistance (MDR) Transporters

Complementing CSC persistence, multidrug resistance (MDR) transporters represent another cornerstone of TNBC refractoriness, facilitating active extrusion of chemotherapeutic agents from intracellular compartments. TNBC cells frequently overexpress ATP-binding cassette (ABC) family transporters, notably P-glycoprotein (P-gp/ABCB1), which pumps out substrates including anthracyclines, taxanes, and platinum compounds, thereby attenuating intracellular drug levels to subtherapeutic concentrations and conferring cross-resistance to multiple classes [52,53]. In TNBC cohorts, ABCB1 upregulation correlates with poorer response to neoadjuvant therapy (pCR rates < 20% vs. >50% in low-expressors) and reduced overall survival (HR 1.8; 95% CI 1.2–2.7), as ABCB1 expression is induced by stress signals like NF-κB activation in the tumor microenvironment (TME) [54,55]. Beyond ABCB1, multidrug resistance-associated protein 1 (MRP1/ABCC1) and breast cancer resistance protein (BCRP/ABCG2) synergize to broaden the efflux spectrum, with ABCG2 particularly implicated in resistance to irinotecan derivatives like SN-38 in ADC contexts [41]. This transporter-mediated efflux is dynamically regulated by epigenetic modifications, such as ABCB1 promoter hypomethylation in resistant clones, underscoring the need for transporter inhibitors like tariquidar, which have shown promise in preclinical TNBC models to restore drug sensitivity [56] (Figure 2).

TNBC exhibits multiple interconnected resistance mechanisms. (1) CSC enrichment: Dormant CD44+/CD24− cancer stem cells possess self-renewal capacity and intrinsic chemoresistance, enabling survival during therapy and tumor recurrence. (2) MDR transporters: P-glycoprotein (ABCB1) and BCRP (ABCG2) actively efflux chemotherapeutic agents, reducing intracellular drug accumulation. (3) TME barrier: Dense collagen/fibrosis impedes drug penetration and immune infiltration, while hypoxia-driven HIF-1α activation promotes chemoresistance. (4) Metabolic plasticity: TNBC cells switch between glycolysis, OXPHOS, and FAO to maintain viability under therapeutic stress. These mechanisms collectively necessitate combination strategies targeting multiple resistance pathways.

### 3.3. Metabolic Plasticity (OXPHOS & Fatty Acid Oxidation)

Furthermore, metabolic plasticity emerges as a pivotal adaptive strategy in drug-resistant TNBC, allowing cells to rewire energy production pathways to sustain survival under therapeutic stress. While bulk TNBC tumors predominantly favor aerobic glycolysis (Warburg effect), resistant subpopulations shift toward oxidative phosphorylation (OXPHOS) and fatty acid oxidation (FAO) to generate ATP more efficiently in nutrient-scarce or hypoxic TME [57,58]. This transition is orchestrated by peroxisome proliferator-activated receptor gamma coactivator 1-alpha (PGC-1α), which upregulates mitochondrial biogenesis and FAO enzymes like carnitine palmitoyltransferase 1 (CPT1), enabling FAO-dependent OXPHOS to fuel DNA repair and redox homeostasis during chemotherapy exposure [59,60]. In TNBC PDX models, FAO inhibition with etomoxir sensitizes resistant cells to doxorubicin by depleting OXPHOS reserves, reducing tumor regrowth by 60% [61]. Similarly, glutaminolysis supports anaplerotic replenishment of TCA cycle intermediates for OXPHOS, with glutaminase (GLS) inhibition synergizing with taxanes to overcome resistance in GLS-high TNBC lines [62]. This metabolic flexibility not only confers intrinsic resistance but also remodels the TME by secreting lactate [63] and exosomes [64] that suppress immune infiltration, perpetuating a vicious cycle of recurrence (Figure 2). Although OXPHOS inhibitors such as IACS-010759 demonstrated robust preclinical efficacy, their clinical translation has been stymied by an unfavorable therapeutic index and dose-limiting toxicities, notably neuropathy and cardiotoxicity. This underscores the imperative for next-generation agents with superior tumor selectivity. Concurrently, the clinical interrogation of metabolic vulnerabilities persists, exemplified by ongoing trials (e.g., NCT02871700) evaluating FAO inhibitors in TNBC patients.

### 3.4. TME Remodeling (Fibrosis & Hypoxia)

The TME further entrenches resistance through physical and immunological barricades that impair drug penetration and effector cell access. Dense peritumoral fibrosis, driven by cancer-associated fibroblasts (CAFs) secreting collagen I and III, creates a stiff extracellular matrix (ECM) that compresses vasculature and elevates interstitial fluid pressure, reducing intratumoral drug delivery by up to 90% in fibrotic TNBC lesions [65,66]. In TNBC, fibrosis correlates with mesenchymal subtype enrichment and poor pCR rates (<15% vs. 40% in low-fibrosis), as lysyl oxidase (LOX) cross-linking stiffens ECM to promote EMT and CSC maintenance [67]. Concurrently, chronic hypoxia—prevalent in 70% of TNBC tumors due to aberrant angiogenesis—stabilizes HIF-1α, which transcriptionally activates VEGF for neovascularization while suppressing apoptosis and enhancing glycolysis to fuel metastasis [68]. Hypoxic TME also fosters immune evasion by recruiting regulatory T cells (Tregs) and myeloid-derived suppressor cells (MDSCs) via CCL2 and CXCL12 chemokines, limiting cytotoxic T-cell infiltration and blunting ICI efficacy [69,70]. While KEYNOTE-355 demonstrated the efficacy of pembrolizumab in PD-L1+ TNBC, a significant proportion of patients do not respond [24]. Hypoxia limits T cell function and blunts the efficacy of checkpoint blockade [71,72], a phenomenon also observed in clinical non-responders [73] and preclinical studies suggest that Hypoxic zones in tumors resist T cell infiltration, and reducing hypoxia (e.g., with TH-302) has been shown to restore sensitivity to checkpoint blockade [72]. These TME-driven barriers underscore the imperative for stromal-targeting strategies, such as LOX inhibitors or hypoxia-activated prodrugs, to dismantle the fibrotic-hypoxic fortress and potentiate systemic therapies [74] (Figure 2).

Collectively, these interconnected resistance axes—CSCs, MDR transporters, metabolic plasticity, and TME remodeling—form a resilient network that not only perpetuates TNBC recurrence but also highlights opportunities for combinatorial interventions. Emerging paradigms integrate CSC-depleting agents (e.g., salinomycin [75] with transporter inhibitors and metabolic modulators (e.g., IACS-010759 for OXPHOS [76]) alongside TME normalizers (e.g., bevacizumab), a strategy supported by concepts of vascular normalization [77]. Future research leveraging single-cell multi-omics will further delineate these dynamics, paving the way for precision strategies to uproot the seeds of relapse.

## 4. Targeting Canonical Signaling and DNA Damage Response

### 4.1. Lessons from the PI3K/AKT/mTOR Pathway

#### 4.1.1. Clinical Setbacks: Why Inhibitors Failed

Hyperactivation of the PI3K/AKT/mTOR pathway occurs in approximately 35–50% of TNBC cases, primarily driven by activating mutations in PIK3CA (15–25%) and AKT1 (5–10%), or the loss of PTEN (30–50%), leading to tumor aggressiveness and therapeutic resistance [4,5]. Unlike hormone receptor-positive (HR+) breast cancer, targeting this pathway in TNBC is notoriously challenging due to intratumoral heterogeneity, rapid compensatory signaling via alternative pathways such as RAS/MAPK, and high toxicity profiles including hyperglycemia and rash [78]. Furthermore, pathway reactivation triggered by PI3K inhibition—through insulin feedback loops [79] or the release of mTORC1-S6K1 inhibition [80]—remains a major factor limiting the efficacy of monotherapies.

**Figure 3:**
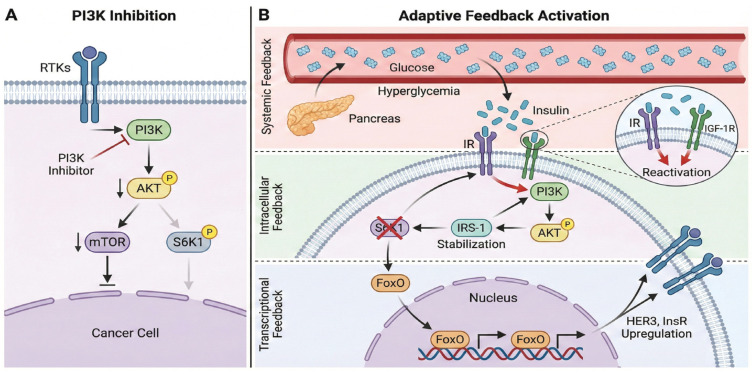
PI3K/AKT Pathway Inhibition and Adaptive Feedback Resistance Mechanisms. (**A**) PI3K/AKT Pathway Inhibition. Ligand binding to Receptor Tyrosine Kinases (RTKs) initiates the PI3K/AKT/mTOR/S6K1 signaling cascade. Treatment with PI3K inhibitors blocks this transduction, leading to the suppression of downstream effectors AKT, mTOR, and S6K1. (**B**) Adaptive Feedback Activation. Therapeutic efficacy is limited by three compensatory mechanisms that drive resistance: (1) Systemic Feedback: PI3K inhibition blocks glucose uptake in peripheral tissues, inducing hyperglycemia. This physiological stress triggers a surge of insulin release from the pancreas. High levels of insulin bind to insulin receptors and IGF-1R on the tumor cell surface, forcefully reactivating the pathway. (2) Intracellular Feedback: The loss of S6K1 activity relieves the negative feedback loop on IRS-1. This leads to IRS-1 stabilization, which in turn enhances upstream signaling input to PI3K and AKT. (3) Transcriptional Feedback: Inhibition of AKT prevents the phosphorylation of FoxO transcription factors, allowing their nuclear translocation. Once in the nucleus, FoxO drives the transcriptional upregulation of RTKs (e.g., HER3, InsR), increasing receptor density on the cell membrane and restoring signaling flux.

Recent clinical trials have highlighted these limitations. The pan-AKT inhibitor capivasertib, evaluated in metastatic TNBC settings (e.g., PAKT and subsequent trials), improved progression-free survival (PFS) in PIK3CA/AKT1/PTEN-altered subgroups but has faced challenges in demonstrating a significant extension in overall survival (OS) across broader populations [81]. Similarly, Roche’s ipatasertib failed to demonstrate an OS benefit in the IPATunity-130 trial despite PFS improvements in the biomarker-positive group, and its development for TNBC was discontinued following the IPATunity-170 trial due to immune-related toxicity issues [82]. These consecutive clinical failures underscore the need for strategies beyond simple enzymatic inhibition (Figure 3).

#### 4.1.2. Mechanisms of Resistance: Feedback Loops and Compensatory Signaling

First, systemic metabolic feedback plays a critical role. The inhibition of PI3K/AKT blocks glucose uptake in peripheral tissues (skeletal muscle and liver), inducing acute hyperglycemia. This physiological stress triggers a compensatory surge in insulin release from the pancreas. The supraphysiological levels of insulin then bind to insulin receptors on the tumor cell surface, forcefully reactivating the PI3K signaling pathway and negating the drug’s effect [79].

Second, intracellular signaling rewiring occurs through the relief of negative feedback loops. Under normal conditions, the mTORC1-S6K1 axis phosphorylates and degrades IRS-1, keeping upstream signaling in check. However, the inhibition of this pathway releases this brake, leading to the stabilization of IRS-1 and paradoxical pathway reactivation driven by upstream receptor tyrosine kinases [80].

Third, transcriptional adaptation further fuels resistance. AKT inhibition prevents the phosphorylation of FoxO transcription factors (FoxO1/3a), allowing them to translocate into the nucleus. Once nuclear, FoxO drives the transcriptional upregulation of multiple RTKs, such as HER3, IGF-1R, and InsR. This “rebound” expression creates a new signaling input that bypasses the initial blockade [83].

Furthermore, TNBC cells exhibit profound plasticity, rapidly adapting by upregulating parallel bypass signaling pathways, such as RAS/MAPK or Wnt/β-catenin [78]. Beyond signaling, cells also engage stress-response survival mechanisms; for instance, PI3K/mTOR inhibition induces cytoprotective autophagy, allowing cancer cells to recycle intracellular components and maintain metabolic homeostasis under therapeutic stress [84]. Finally, epigenetic reprogramming involving BRD4-mediated enhancer remodeling has been identified as a key driver of transcriptional plasticity that sustains cell survival despite kinase inhibition [85].

### 4.2. Synthetic Lethality: Cell Cycle and DDR Kinases

#### 4.2.1. Targeting the G2/M Checkpoint: WEE1, ATR and Beyond

Given the high frequency of TP53 mutations (>80%) and the prevalence of intrinsic replication stress in TNBC, kinases regulating the G2/M checkpoint and DNA damage response (DDR) have emerged as highly attractive targets for synthetic lethality [10]. In TP53-mutant TNBC, the G1/S checkpoint is functionally defective, forcing tumor cells to rely heavily on the G2/M checkpoint—controlled primarily by WEE1 and ATR—to repair DNA damage before mitosis [86,87]. Without this pause, cells undergo premature mitotic entry, leading to mitotic catastrophe and cell death.

##### WEE1 Inhibition

Phase 2 clinical data indicates that the WEE1 inhibitor Adavosertib (AZD1775) is particularly effective in tumors harboring CCNE1 (Cyclin E1) amplification. CCNE1 overexpression drives aberrant S-phase entry and induces massive replication stress, rendering cells acutely hypersensitive to WEE1 inhibition [86]. Furthermore, recent studies suggest that PKMYT1, a kinase structurally related to WEE1 that phosphorylates CDK1, represents a distinct and promising therapeutic target. PKMYT1 inhibition via RP-6306 has shown potent synthetic lethality in CCNE1-amplified tumors by forcing cells with replication stress to skip the G2 checkpoint, offering a strategy to overcome potential resistance to WEE1 inhibitors [88].

##### ATR Inhibition

The ATR kinase serves as the master regulator of the replication stress response. In the plasmaMATCH trial, the ATR inhibitor Ceralasertib combined with the PARP inhibitor Olaparib demonstrated promising activity in TNBC patients with ATM loss or functional Homologous Recombination Deficiency (HRD), even in the absence of BRCA mutations [87]. Beyond combinations, monotherapy with next-generation ATR inhibitors like Camonsertib (RP-3500) is showing efficacy in tumors with ATM deficiency. ATM loss creates a dependency on ATR for survival; thus, blocking ATR in these tumors disrupts the repair of stalled replication forks, leading to double-strand breaks and apoptosis [89].

##### Challenges and Optimization

**Figure 4:**
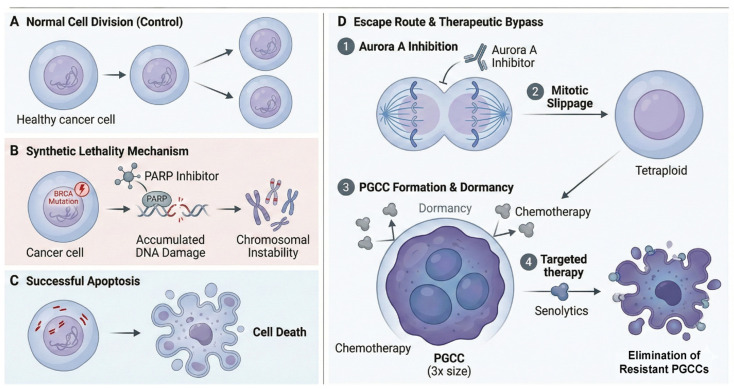
Synthetic Lethality versus Escape via Polyploidy in TNBC. (**A**) Normal Mitosis. Represents a proliferation-competent cancer cell completing mitosis to produce two identical daughter cells. (**B**) Synthetic Lethality. Depicts the mechanism of synthetic lethality in BRCA-mutant (HRD) cells. Pharmacological inhibition of PARP blocks single-strand break repair, leading to the generation of catastrophic double-strand breaks and severe replication stress. (**C**) Apoptotic Outcome. Following the events in (**B**), the massive burden of DNA damage activates apoptotic signaling pathways, resulting in cell elimination. (**D**) Escape Mechanism and Therapeutic Bypass. Stepwise progression of resistance to Aurora A kinase inhibitors and a proposed combination strategy. (1) Mitotic Arrest: Aurora A inhibition compromises spindle assembly, arresting cells in mitosis. (2) Mitotic Slippage: Arrested cells bypass cytokinesis and revert to an interphase state without dividing, resulting in tetraploidy. (3) Formation of Dormant PGCCs: These tetraploid cells evolve into multinucleated Polyploid Giant Cancer Cells (PGCCs), characterized by significant hypertrophy and entry into a therapy-resistant dormant phase. (4) Senolytic Therapy: Utilizing senolytics to specifically target and eliminate the senescent-like PGCC population, thereby preventing disease recurrence.

While promising, G2/M checkpoint inhibitors face hurdles regarding toxicity, particularly myelosuppression and gastrointestinal adverse events, which often necessitate dose reductions. Current efforts focus on optimizing dosing schedules (e.g., intermittent dosing) and identifying robust biomarkers beyond TP53 and CCNE1—such as SLFN11 expression—to predict sensitivity and widen the therapeutic window [90] (Figure 4).

#### 4.2.2. CDK4/6 Limitations and New Opportunities

While CDK4/6 inhibitors have revolutionized HR+ breast cancer treatment, their efficacy in TNBC remains limited due to the frequent functional loss of the RB1 tumor suppressor gene, particularly in basal-like subtypes [10,11]. Because these inhibitors require functional RB1 to arrest the cell cycle, RB1-deficient TNBC cells render the drug target irrelevant and proliferate unchecked [10]. To leverage this mechanism, Trilaciclib (Cosela) was developed as a myelopreservation therapy rather than a direct anticancer agent [91]. By transiently arresting hematopoietic stem cells, it aims to shield the bone marrow from chemotherapy toxicity while sparing RB1-deficient tumor cells, which remain unaffected by the blockade [91].

However, the pivotal Phase 3 PRESERVE-2 trial reported in 2025 failed to meet its primary endpoint of Overall Survival (OS) [92]. Although Trilaciclib successfully achieved its pharmacodynamic goal of reducing severe neutropenia, this supportive care benefit did not translate into an extension of life [92]. These findings highlight that “shielding the host” is insufficient to overcome aggressive metastatic TNBC without a concurrent anti-tumor driver. Notably, the unsuccessful outcome of the Phase 3 PRESERVE-2 trial illuminates a pivotal therapeutic paradigm: in aggressive malignancies such as metastatic TNBC, strategies exclusively targeting myelopreservation fail to translate into Overall Survival (OS) benefits in the absence of concomitant, direct anti-tumor efficacy. Future CDK4/6 strategies must therefore pivot to RB1-intact subgroups, such as the LAR subtype, or explore immune combinations [91].

## 5. Expanding the Horizon: Mitotic and Undrugged Kinases

### 5.1. The New Frontier: Mitotic Kinases

Directly targeting the mitotic machinery has witnessed a renaissance, shifting from broad-spectrum cytotoxic effects to precision interference with mitotic checkpoints. This resurgence is driven by the development of highly selective inhibitors with improved pharmacokinetic profiles that minimize the dose-limiting toxicities, such as neutropenia, that plagued earlier generations.

#### 5.1.1. PLK1: Mitotic Catastrophe Inducers

Polo-like Kinase 1 (PLK1) serves as a master regulator of mitosis, orchestrating centrosome maturation, spindle assembly, and cytokinesis [93]. In TNBC, PLK1 is frequently overexpressed compared to other breast cancer subtypes and correlates with poor overall survival, making it a high-value target for therapeutic intervention [94]. Unlike first-generation inhibitors (e.g., volasertib) which were hampered by poor specificity and high rates of severe neutropenia [95], Onvansertib has emerged as a third-generation, highly selective oral PLK1 inhibitor with a manageable safety profile [96,97].

Recent Phase 1b/2 clinical data presented in 2024 and 2025 have highlighted the potent synergy between Onvansertib and taxanes. Mechanistically, while taxanes stabilize microtubules to arrest cells in mitosis, PLK1 inhibition prevents the proper satisfaction of the spindle assembly checkpoint and cytokinesis [97]. This dual targeting forces cells to exit mitosis prematurely without dividing—a state known as mitotic catastrophe—thereby overcoming taxane resistance [96]. The trial results demonstrated a 40% Objective Response Rate (ORR) in patients with metastatic TNBC who had previously progressed on multiple lines of therapy, including taxanes and anthracyclines [96]. This response rate far exceeds historical controls for standard-of-care chemotherapy in the resistant setting, suggesting that Onvansertib can re-sensitize resistant tumors to taxanes by exploiting the cell’s reliance on PLK1 for mitotic exit [94,96].

#### 5.1.2. TTK and Spindle Assembly Checkpoint Inhibitors

In parallel, targeting the spindle assembly checkpoint (SAC) via TTK (also known as MPS1) represents a strategy to exploit the pervasive genomic instability characteristic of TNBC [11]. The SAC acts as a “quality control” mechanism during cell division, ensuring chromosomes are correctly aligned before segregation. Inhibiting TTK overrides this checkpoint, causing tumor cells to divide with unaligned chromosomes, leading to severe aneuploidy and subsequent cell death [98].

CFI-402257, a potent oral TTK inhibitor, has shown robust efficacy in preclinical models and early-phase trials. Notably, a study published in 2024 revealed a novel synthetic lethal interaction between TTK inhibition and metabolic stress [98]. The research demonstrated that energy stress, mediated by AMP-activated protein kinase (AMPK) activation, sensitizes TNBC cells to TTK inhibition. Under conditions of metabolic stress, the combined disruption of the mitotic checkpoint by CFI-402257 leads to an accelerated apoptotic response in TNBC cells with high chromosomal instability (CIN), while sparing normal cells [98]. This suggests that combining TTK inhibitors with metabolic modulators (e.g., AMPK agonists like metformin) could be a viable therapeutic strategy for metabolically active TNBC subtypes.

#### 5.1.3. Aurora Kinase Failures and PGCC Formation

However, targeting mitotic kinases is not without pitfalls, and the clinical failure of Aurora Kinase inhibitors offers critical lessons. Despite a strong preclinical rationale where Aurora A inhibition disrupted spindle pole formation and induced apoptosis, the selective inhibitor Alisertib failed to demonstrate a survival benefit in randomized clinical trials for metastatic breast cancer [99]. While initial studies showed promise, the pivotal randomized trials (e.g., in combination with paclitaxel) revealed that progression-free survival benefits did not translate into overall survival, particularly in the diverse landscape of TNBC, leading to the discontinuation of its broad clinical development [99]. The limited clinical success of Alisertib underscores a counterintuitive biological outcome: the induction of mitotic arrest may fail to trigger apoptosis and instead promote the emergence of Polyploid Giant Cancer Cells (PGCCs), which serve as a dormant seed for tumor relapse.

This discrepancy between preclinical success and clinical failure prompted deep mechanistic interrogation. Recent studies published in 2025 have revealed that instead of undergoing immediate apoptosis as hypothesized, TNBC cells treated with Alisertib engage a survival program known as endoreplication [100]. In this process, cells bypass cytokinesis but continue DNA replication, resulting in the formation of multinucleated Polyploid Giant Cancer Cells (PGCCs) [100]. These PGCCs are not merely dying cells; rather, they enter a dormant, senescent-like state that renders them resistant to conventional chemotherapy and mitotic poisons [101].

Crucially, these giant cells retain the capacity to eventually undergo depolyploidization—a process termed neosis or asymmetric division—and “bud off” small, proliferative progeny [100,101]. These progeny cells often possess enhanced stem-like properties and metabolic flexibility, thereby seeding aggressive disease relapse [100,101]. These findings fundamentally reshape our understanding of resistance to mitotic inhibitors, underscoring the necessity of combining agents like Alisertib with senolytics (drugs that selectively kill senescent cells) or other therapies that can specifically eliminate these dormant PGCCs to prevent recurrence [100].

### 5.2. Unlocking the Undrugged Kinome

The past five years have represented a period of aggressively challenging the “Undrugged Kinome,” a class of kinase targets previously deemed inaccessible for drug development due to their lack of enzymatic activity (pseudokinases) or structural ubiquity causing toxicity. Advances in structural biology and novel modalities like PROTACs and ADCs are now dismantling these barriers.

#### 5.2.1. TNIK: Targeting the Roots of Stemness

The past five years have also represented a period of challenging the “Undrugged Kinome,” a class of targets previously deemed inaccessible for drug development. TNIK, a key effector of Wnt signaling, is currently under investigation as a therapeutic target for preventing recurrence due to its critical role in maintaining CSCs [102,103]. TNIK functions as an essential activator of the Wnt/β-catenin signaling pathway, not merely as a passive downstream effector but as a key amplifier that physically interacts with TCF4 and β-catenin to drive the transcription of stemness-associated genes [102].

Recent preclinical data indicates that TNBC cells surviving neoadjuvant chemotherapy exhibit significantly elevated TNIK activity, suggesting its role in driving disease recurrence [103]. Unlike broad-spectrum kinase inhibitors, specific inhibition of TNIK (e.g., with the small molecule NCB-0846) has been shown to disrupt the Wnt signaling axis, selectively eliminating the CD44+/CD24− CSC population that fuels relapse [102]. Furthermore, latest studies suggest a synthetic lethal interaction between TNIK inhibition and immune checkpoint blockade. By downregulating Wnt-driven immunosuppression—which is known to exclude T-cells from the tumor microenvironment—TNIK inhibitors may sensitize “cold” TNBC tumors to anti-PD-1 therapies [104], positioning TNIK as a dual-purpose target for eradicating stemness and enhancing immunity. It is important to note, however, that TNIK targeting is currently limited to preclinical investigation. The translation of the robust anti-stemness effects observed in PDX models into clinical efficacy and an acceptable safety profile necessitates rigorous validation in future trials.

#### 5.2.2. PTK7: Turning a Pseudokinase into a Precision Target

PTK7 (Protein Tyrosine Kinase 7) represents a paradigm shift in kinase drug discovery. As a pseudokinase lacking a catalytic active site, PTK7 was historically considered “undruggable” by traditional ATP-competitive inhibitors. However, its overexpression in TNBC and its pivotal role in the Wnt/Planar Cell Polarity (PCP) pathway—which drives EMT and metastasis—make it a high-priority target [105].

Since catalytic inhibition is impossible, recent strategies have pivoted to exploiting PTK7 as a surface anchor for payload delivery. Cofetuzumab pelidotin, a PTK7-directed Antibody–Drug Conjugate (ADC), has demonstrated clinical proof-of-concept, delivering a cytotoxic payload specifically to PTK7-expressing TNBC cells [106]. Moreover, 2024 reviews highlight the emergence of targeted protein degraders (PROTACs) and bispecific antibodies that physically remove PTK7 protein from the cell surface or redirect T-cells to PTK7+ tumors [105]. These approaches bypass the need for enzymatic inhibition, effectively turning the structural “defect” of this pseudokinase into a therapeutic handle for precise tumor ablation [105]. PTK7-directed ADC strategies emerge as a compelling therapeutic paradigm for patient subgroups defined by diminished TROP2 or HER2 antigen density, or those exhibiting acquired resistance to standard-of-care agents such as Sacituzumab Govitecan or Trastuzumab Deruxtecan, particularly within Wnt-driven TNBC phenotypes.

#### 5.2.3. PAK4: Overcoming Immune Exclusion and Structural Homology

Similarly, PAK4 (p21-activated kinase 4) has long been recognized as a driver of stemness, cell migration, and, crucially, immune evasion in TNBC. However, its development was stalled by severe toxicity concerns due to the high structural homology of its ATP-binding pocket with other essential kinases (PAK1-3) [107].

Next-generation efforts are now actively overcoming these hurdles through the development of allosteric inhibitors and highly selective degraders. A landmark study revealed that PAK4 inhibition does more than arrest cell growth; it reverses the “immune exclusion” phenotype. PAK4 normally stabilizes β-catenin to suppress T-cell infiltration; thus, selectively inhibiting PAK4 normalizes the tumor vascular network and facilitates the influx of CD8+ T cells, sensitizing resistant TNBC tumors to PD-1 blockade [108]. Building on this, novel agents like KPT-9274 are currently under investigation, aiming to provide a dual strike against TNBC by disrupting metabolic dependence (via NAMPT inhibition) and reversing immune suppression, thereby validating PAK4 as a linchpin target for next-generation combinatorial immunotherapy [107]. Crucially, the therapeutic utility of selective PAK4 inhibition may be compromised by adaptive feedback loops, wherein the compensatory upregulation of alternative isoforms (e.g., PAK1) precipitates resistance. This underscores the rationale for pan-PAK targeting or synergistic combinatorial regimens to achieve durable therapeutic responses.

## 6. Technological Evolution and Future Paradigms

### 6.1. Beyond Inhibition: The Rise of PROTAC Technology

**Figure 5:**
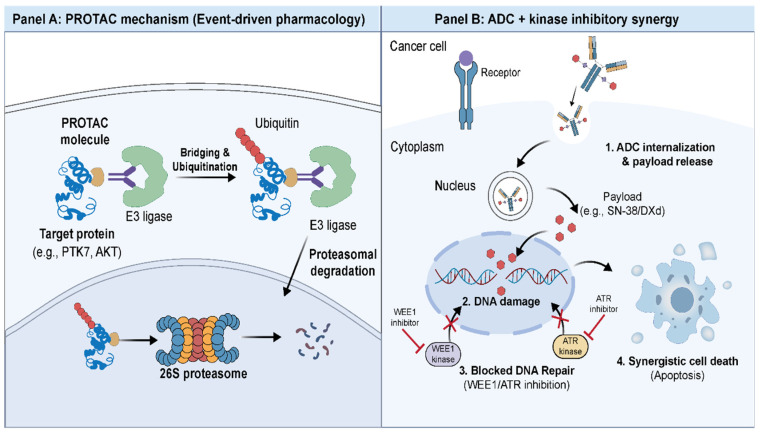
PROTAC-Mediated Targeted Protein Degradation and ADC-Kinase Inhibitor Combination Therapy. Panel (**A**) PROTAC Mechanism. PROTACs are bifunctional molecules that simultaneously bind a target protein (e.g., PTK7, AKT) and an E3 ubiquitin ligase, forming a ternary complex. This proximity-induced ubiquitination marks the target protein for proteasomal degradation. Unlike conventional inhibitors, PROTACs function catalytically, enabling substoichiometric dosing and degradation of proteins lacking enzymatic active sites. Panel (**B**) ADC + Kinase Inhibitor Synergy. (1) ADCs deliver cytotoxic payloads (SN-38/DXd) selectively to tumor cells via receptor-mediated internalization. (2) Released topoisomerase I inhibitors cause DNA strand breaks. (3) WEE1 and ATR kinase inhibitors block the DNA damage response and G2/M checkpoint, preventing repair. (4) The combination of ADC-induced DNA damage with blocked repair mechanisms results in syn-ergistic apoptotic cell death, exploiting tumor-selective delivery while preventing adaptive re-sistance.

To overcome the inherent limitations associated with traditional small-molecule inhibitors—such as the requirement for high occupancy of the active site and the inability to target proteins lacking enzymatic pockets—drug development trends since 2021 have shifted aggressively toward targeted protein degradation (PROTAC) technology. Unlike inhibitors which merely block protein function (occupancy-driven), PROTACs utilize the cell’s ubiquitin-proteasome system (UPS) to physically eliminate the target protein (event-driven) [109] (Figure 5). This mechanism offers a distinct advantage: a single PROTAC molecule can function catalytically, destroying multiple copies of the target, thereby achieving efficacy at lower doses and overcoming resistance caused by protein overexpression or mutations.

For “undruggable” targets like PTK7 (which lacks catalytic activity) or PAK4 (where kinase selectivity is structurally difficult to achieve), PROTACs offer an effective alternative by targeting the whole protein for destruction rather than inhibiting a specific domain. Recent breakthroughs in 2024 have demonstrated the power of this approach in targeting AKT. While traditional AKT inhibitors often trigger feedback loops that lead to pathway reactivation, next-generation degraders (such as MS-21) capable of completely eliminating AKT protein have demonstrated potent efficacy in resistant TNBC cell lines. By removing the protein entirely, these degraders dismantle both the catalytic and scaffolding functions of AKT, preventing the compensatory signaling that typically renders inhibitors ineffective [110].

Furthermore, 2025 has seen the convergence of degradation technology with nanotechnology, exemplified by platforms such as “Nano-PROTACs” or photodynamic degraders. Researchers have developed systems that combine TNBC-targeting ligands with PROTAC payloads, sometimes activated by light (photodynamic therapy). This approach induces both tumor-specific degradation of resistance drivers and physical destruction of the tumor architecture via reactive oxygen species (ROS), presenting a novel therapeutic paradigm that bypasses classical resistance mechanisms entirely [111].

Panel A: PROTAC Mechanism. PROTACs are bifunctional molecules that simultaneously bind a target protein (e.g., PTK7, AKT) and an E3 ubiquitin ligase, forming a ternary complex. This proximity-induced ubiquitination marks the target protein for proteasomal degradation. Unlike conventional inhibitors, PROTACs function catalytically, enabling substoichiometric dosing and degradation of proteins lacking enzymatic active sites. Panel B: ADC + Kinase Inhibitor Synergy. (1) ADCs deliver cytotoxic payloads (SN-38/DXd) selectively to tumor cells via receptor-mediated internalization. (2) Released topoisomerase I inhibitors cause DNA strand breaks. (3) WEE1 and ATR kinase inhibitors block the DNA damage response and G2/M checkpoint, preventing repair. (4) The combination of ADC-induced DNA damage with blocked repair mechanisms results in synergistic apoptotic cell death, exploiting tumor-selective delivery while preventing adaptive resistance.

### 6.2. Strategic Combinations: Kinase Inhibitors as ADC Partners

As of 2025, the most promising clinical strategy for overcoming drug resistance involves utilizing kinase inhibitors not merely as monotherapies, but as potent enhancers of ADCs. The rationale lies in the mechanism of action of ADCs like Sacituzumab Govitecan (SG) or Trastuzumab Deruxtecan (T-DXd), which deliver DNA-damaging payloads (topoisomerase I inhibitors: SN-38 or DXd) directly to tumor cells (Figure 5) [26,43].

When resistant cancer cells are hit by these cytotoxic payloads, they do not die immediately; instead, they activate adaptive DDR pathways to repair the induced double-strand breaks and survive [112]. This is where kinase inhibitors play a critical role. Concurrent administration of ATR, WEE1, or DNA-PK inhibitors can block these survival/repair mechanisms. For instance, recent preclinical studies have demonstrated that blocking WEE1 prevents the G2/M arrest that cells need to repair SN-38-induced damage. This forces cells with damaged DNA to enter mitosis prematurely, leading to a specific form of cell death known as “checkpoint abrogation” or “mitotic catastrophe” [113]. This synergy is currently being investigated in innovative triplet regimens. Exemplifying the bench-to-bedside translation of this paradigm, an ongoing clinical trial (NCT05154240) is currently interrogating the synergistic potential of Sacituzumab Govitecan combined with the ATR inhibitor Berzosertib—a strategy predicated on exploiting preclinical synthetic lethality vulnerabilities.

The BEGONIA trial (Arm 6) has already shown the potential of combining an ADC (T-DXd) with immunotherapy (Durvalumab) to enhance efficacy [114]. Building on this, next-generation arms are exploring the addition of DDR kinase inhibitors to this backbone. These triplet strategies aim to simultaneously address three pillars of treatment: (1) precise drug delivery via ADCs, (2) blockade of repair mechanisms via kinase inhibitors, and (3) immune activation via checkpoint blockade [115]. This multi-modal approach offers a comprehensive blueprint to conquer refractory TNBC by cutting off escape routes at the DNA repair, metabolic, and immunological levels [113,114].

## 7. Conclusions

The 2021–2025 era marks a definitive paradigm shift in TNBC therapy, from broad cytotoxicity to the precision targeting of resistance mechanisms, specifically CSCs and metabolic plasticity. The clinical setbacks of PI3K/AKT inhibitors, driven by adaptive feedback loops, have necessitated a pivot toward synthetic lethality, focusing on G2/M checkpoint regulators (WEE1, ATR) and mitotic kinases like PLK1. Currently, the most transformative advancements lie in unlocking the “undruggable” kinome via PROTAC and utilizing kinase inhibitors as potent enhancers of ADCs to block DNA repair. Ultimately, the future of TNBC treatment depends on multi-omics-guided combinatorial strategies that integrate these novel modalities to dismantle therapeutic resistance and improve patient survival.

## Data Availability

No new data were created or analyzed in this study. Data sharing is not applicable to this article.

## Abbreviations

ADC	Antibody-Drug Conjugate
AKT	Protein Kinase B (PKB)
AR	Androgen Receptor
ATM	Ataxia Telangiectasia Mutated
ATR	Ataxia Telangiectasia and Rad3-related
BL1/2	Basal-like 1/2
CCNE1	Cyclin E1
CDK	Cyclin-Dependent Kinase
CI	Confidence Interval
CPS	Combined Positive Score
Dato-DXd	Datopotamab Deruxtecan
DDR	DNA Damage Response
DYRK1A	Dual Specificity Tyrosine Phosphorylation Regulated Kinase 1A
ER	Estrogen Receptor
gBRCA	Germline Breast Cancer Gene (BRCA1/2)
HER2	Human Epidermal Growth Factor Receptor 2
HR	Hazard Ratio
HRD	Homologous Recombination Deficiency
IM	Immunomodulatory
IR	Insulin Receptor
IRS-1	Insulin Receptor Substrate-1
LAR	Luminal Androgen Receptor
M	Mesenchymal
MAPK	Mitogen-Activated Protein Kinase
mTOR	Mammalian Target of Rapamycin
mTNBC	Metastatic Triple-Negative Breast Cancer
NTRK	Neurotrophic Tyrosine Receptor Kinase
ORR	Objective Response Rate
OS	Overall Survival
PARP	Poly (ADP-ribose) Polymerase
PD-L1	Programmed Death-Ligand 1
PFS	Progression-Free Survival
PGCCs	Polyploid Giant Cancer Cells
PI3K	Phosphatidylinositol 3-Kinase
PLK1	Polo-like Kinase 1
PR	Progesterone Receptor
PTEN	Phosphatase and Tensin Homolog
RB1	Retinoblastoma 1
RTK	Receptor Tyrosine Kinase
S6K1	Ribosomal Protein S6 Kinase Beta-1
SG	Sacituzumab Govitecan
TNBC	Triple-Negative Breast Cancer
T-DXd	Trastuzumab Deruxtecan
TTK	Threonine Tyrosine Kinase (also known as Mps1)
VRK1	Vaccinia Related Kinase 1
WEE1	WEE1 G2 Checkpoint Kinase
